# Climate change alters slug abundance but not herbivory in a temperate grassland

**DOI:** 10.1371/journal.pone.0283128

**Published:** 2023-03-14

**Authors:** Daniel Weber, Rebecca K. McGrail, A. Elizabeth Carlisle, James D. Harwood, Rebecca L. McCulley

**Affiliations:** 1 Department of Plant & Soil Sciences, University of Kentucky, Lexington, KY, United States of America; 2 Department of Entomology, University of Kentucky, Lexington, KY, United States of America; Helmholtz Centre for Environmental Research - UFZ, GERMANY

## Abstract

Climate change will significantly impact the world’s ecosystems, in part by altering species interactions and ecological processes, such as herbivory and plant community dynamics, which may impact forage quality and ecosystem production. Yet relatively few field experimental manipulations assessing all of these parameters have been performed to date. To help fill this knowledge gap, we evaluated the effects of increased temperature (+3°C day and night, year-round) and precipitation (+30% of mean annual rainfall) on slug herbivory and abundance and plant community dynamics biweekly in a pasture located in central Kentucky, U.S.A. Warming increased slug abundance once during the winter, likely due to improving conditions for foraging, whereas warming reduced slug abundance at times in late spring, mid-summer, and early fall (from 62–95% reduction depending on month). We found that warming and increased precipitation did not significantly modify slug herbivory at our site, despite altering slug abundance and affecting plant community composition and forage quality. Climate change will alter seasonal patterns of slug abundance through both direct effects on slug biology and indirect effects mediated by changes in the plant community, suggesting that pasture management practices may have to adapt.

## Introduction

Herbivory is an important ecological process in most ecosystems [1, 2], but especially in grasslands which typically co-evolved with considerable levels of mammalian and insect herbivory [3–5]. A lesser studied, but potentially significant, herbivore of grass-dominated ecosystems are molluscs [6, 7]. Gastropods, including slugs, are particularly abundant and problematic in cereal crops where dense stands of annual grasses are grown in cool, wet climates [8–10]. While slugs can have a large negative effect on the yield of row crops [11], it is not always simple to determine the effects of slug or overall mollusc herbivory in pastures or other grasslands where vegetation is more heterogeneous and diverse than croplands [12, 13].

Slugs can both positively and negatively affect plant biomass production depending on a myriad of environmental and biological factors [14–16] and can alter overall plant species composition of vegetative stands through highly selective foraging [17]. For example, in pasture and grassland settings, gastropods have been reported to selectively graze seedlings [16], although this depends on plant species [18], seedling age [18], and nitrogen (N) content [19]. Plant N content is an important factor determining grazing preference for gastropods, with plant material containing greater N preferred when all else is equal [19]. However, as shown by Mattson [19], seedling age appears to be the dominant factor governing palatability for gastropods, due, in part, to young shoots having fewer physical barriers (e.g. production of plant defense compounds) to herbivory in addition to increased N availability. While seedling age may govern grazing selection in monocultures, species identity and abundance can be greater drivers of grazing selection in polycultures [18]. Hanley et al. [18] documented that when multiple species of rosette forbs were grown in polyculture (versus monoculture), rates of herbivory were more dependent on plant species than seedling age. Given these complex factors governing food selection by gastropods, the high species richness and variation of plant age and growth form in most grasslands makes it difficult to predict the effect of slug herbivory on plant community composition [16].

Furthermore, slug herbivory is likely to be impacted by climate change through both direct and indirect interactions [12, 15]. Climate change is expected to increase global average annual temperatures by 1.4–5.8°C and alter hydrological regimes world-wide, in part by altering the timing and magnitude of rain events [20]. Changes in these abiotic parameters could directly and significantly affect slug abundance, habitat suitability and/or geographic range [11, 21, 22]. Gastropods are highly sensitive to abiotic factors, strongly preferring cool, moist conditions [12, 22, 23]. For instance, the urban heat island effect decreases gastropod survival in otherwise suitable environments [23]. Increasing temperatures have resulted in increased mortality of larval [24] and juvenile [25] gastropods and could cause local extinctions of some species. While gastropods typically prefer wetter places in fields, with densities determined primarily by soil moisture, the effect of moisture availability is highly seasonal and changes dramatically from year-to-year [12, 22]. Gastropods have also been shown to expand their range, for example, moving upslope in Swiss National Park—the first such documented case of gastropods with low dispersal capacity moving in response to climate change [26].

Climate change may also have indirect effects on slugs if it alters plant community composition and quantity, quality, or N concentration of the plant material. In an experimental grassland in the United Kingdom, climate manipulations of winter warming and altered summer precipitation were shown to affect slug relative abundance, but this result was attributed primarily to changes in vegetation due to climate treatments [12]. However, in some systems, since slugs can influence the composition of vegetation through seedling herbivory, any direct effects on the slug community from climate change may result in concomitant changes to the plant community [27]. In a factorial study of warming, N addition, and slug exclusion, Moise and Henry [15] found that long-term warming (three years) in an old-field pasture reduced overall plant productivity but that slug exclusion in warmed plots negated the negative effect on biomass of warming alone. These results suggest that the negative effect of warming on grassland productivity was mostly a direct effect of warming increasing slug herbivory [15].

Managed grassland research has shown slugs can have significant effects on plant production and species composition [10, 28]. However, little research on slug herbivory or ecology has been conducted in pastures in North America. It is important to understand what, if any, effects slugs have on pasture productivity in the transitional climatic zone of the United States, which are typically dominated by tall fescue pastures [14 million hectares east of the Mississippi River [29] and >2 million hectares in Kentucky [30] that support animal grazing and other ecosystem services [31]. Unfortunately, not enough is known about slug herbivory and ecology in transitional climatic zone pastures to predict responses to climate change projections for the regions—+1.4–5.8°C mean annual temperature and +10–30% mean annual rainfall by 2100 [21]. To address this gap in knowledge, we measured slug abundance, slug biomass, slug herbivory, plant community composition, plant biomass production, and forage quality in a manipulative, field climate change project in a central Kentucky pasture where temperatures were increased 3°C year-round and growing season precipitation was increased by 30% of the long-term mean.

We hypothesized that slug herbivory would have a measurable negative impact on aboveground plant biomass and that climate change factors would affect slug herbivory, slug abundance, and slug biomass. As reported elsewhere [12, 22], we predicted that slug abundance and biomass would vary over the course of the year and that slug response to the climate treatments would be seasonally dependent. For example, warming might increase slug abundance, biomass, and herbivory during cool spring and autumn months but suppress them during the hot summer. We hypothesized that the combination of warming and additional precipitation would allow slugs to persist and exhibit significant herbivory throughout the summer compared to warmed only plots where they would be less abundant due to their intolerance of drought. However, co-occurring changes in plant community composition, both seasonally and those resulting from the climate change treatments, could modify these responses.

## Materials and methods

### Research site

The experiment was conducted at the University of Kentucky Spindletop Research Farm in Lexington, Kentucky, U.S.A. (38°10’N, 84°49’W). The site was established in an existing climate change project as described in Brosi [32] and Slaughter, Weintraub, and McCulley [33]. After a spring application of glyphosate (Roundup Pro; Monsanto, St Louis, MO, U.S.A.) to remove existing vegetation, the research field was established in the summer of 2008 with ‘Kentucky-31’ tall fescue (*Schedonorus arundinaceus* (Schreb.) Durmort., seeded at a rate of 11.2 kg ha^-1^ pure live seed), ‘Ginger’ Kentucky bluegrass (*Poa pratensis* L., 7.8 kg ha^-1^), ‘Freedom’ red clover (*Trifolium pretense* L., 6.7 kg ha^-1^), and ‘Patriot’ white clover (*Trifolium repens* L., 2.2 kg ha^-1^). Bermudagrass (*Cynodon dactylon* (L.) Pers.) was plugged into the field later in the summer at a rate of 10.76 plugs m^-2^. Soil at the site is deep (>1 m) Maury silt loam (fine, mixed, active, mesic Typic Paleudalfs). Long-term mean annual summer and winter temperatures are 23.8 and 1.6°C, respectively [34], with average annual rainfall of 1137 mm distributed relatively evenly throughout the year. Total annual rainfall (mm) across the study period was 1656 mm (2011), 1019 mm (2012), and 1495 mm (2013) (Kentucky Mesonet; http://www.kymesonet.org/; Lexington, KY, U.S.A.).

During the winter of 2008–2009, twenty 3-m diameter hexagonal plots were established and divided into five replicates of four climate treatments. The climate treatments were ambient conditions (control), +heat (3°C above ambient), +precipitation (+30% of the long-term mean annual rainfall applied during the growing season from rainwater collected on site; +343 mm), and a combination of +heat and +precipitation. Increased temperature was maintained day and night using Salamander infrared heaters (Mor Electrical Heating Associates, Inc., Comstock Park, MI) positioned around the plot following the procedure of Kimball and Conley [35]. Additional rainfall was applied every two weeks throughout the growing season (April-October). Rainfall additions were April +51 mm, May +51 mm, June +62 mm, July +67 mm, August +62 mm, and September +51 mm. The climate treatments began on May 1, 2009 and ran continuously until November 11, 2013.

**Fig 1:**
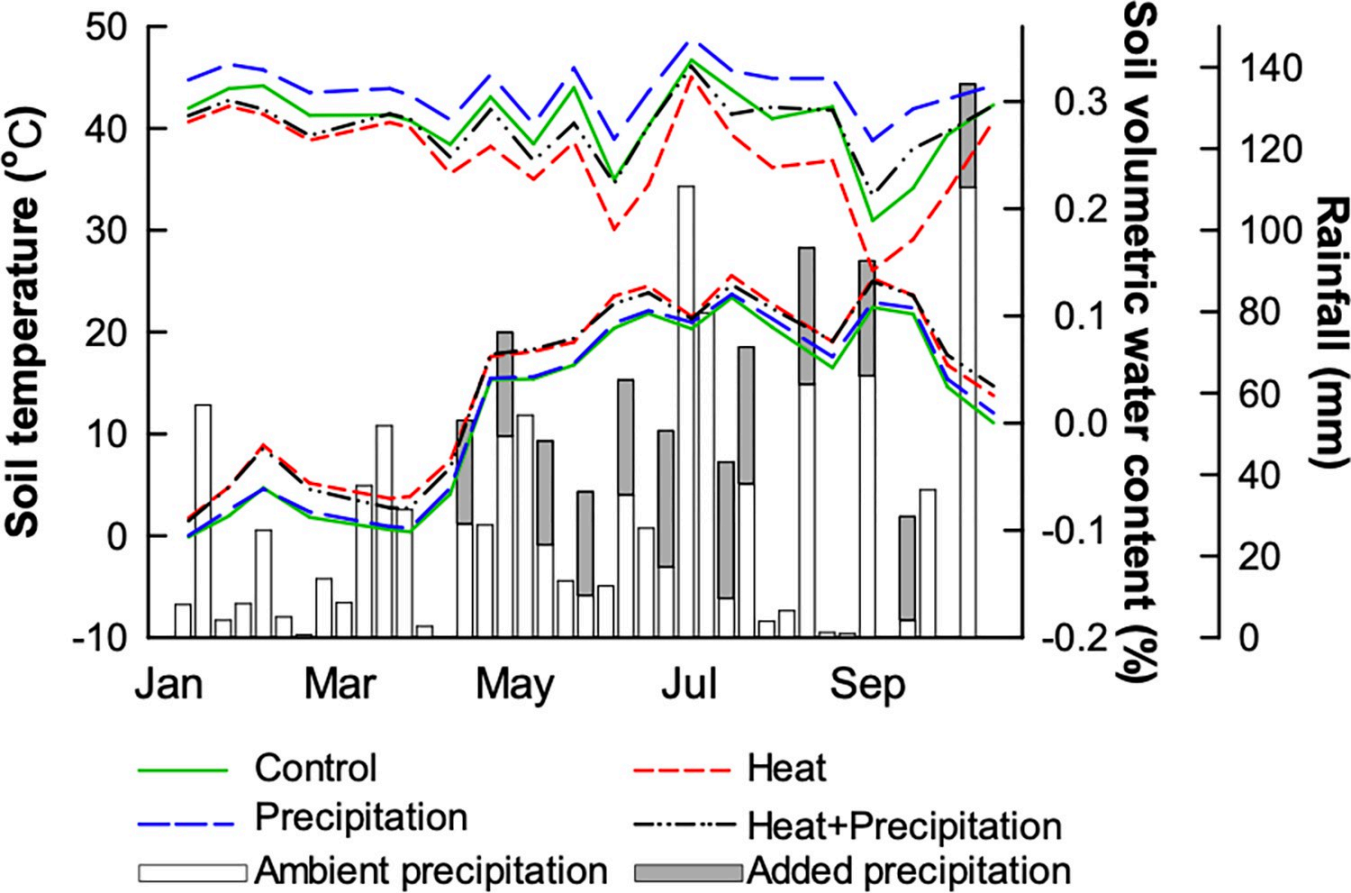
Soil moisture (upper lines) and soil temperature (lower lines; both taken at 5 cm depth) in climate treatments during 2013, as well as ambient precipitation received at the site and amounts of added precipitation applied to the +precipitation treatments.

Each plot was surrounded with aluminum flashing buried 0.5 m deep in the soil. Soil temperature (FW3648 shielded Type T thermocouples; TE Wire, Saddle Brook, NJ, U.S.A) and moisture (CS-616 time domain reflectometers; Campbell Scientific, Logan, UT, U.S.A) at 5 cm below the soil surface were recorded in each plot at 15-minute intervals for the duration of the project. Heated plots were consistently maintained on average +2.8–3°C (air and soil temperature) compared to non-heated plots. Added precipitation plots were consistently wetter than non-irrigated plots by 3–15% volumetric water content with differences between treatments in soil moisture being greatest during the summer and autumn (Fig 1). The field was managed as a hayfield, and all plant material was harvested (6 cm above the soil surface) three times each year and removed offsite. Over the duration of this experiment, harvests occurred on October 5, 2011; May 21, July 30, October 8, 2012; and May 21, August 5, and October 14, 2013.

### Exclosure design

To quantify the effects of slug herbivory on pasture productivity, areas of slug exclusion were constructed and paired with control structures that did not exclude slugs in each experimental plot. The exclosures were modeled after those described by Strauss et al. [36]. Strauss et al. used 50 cm wide rings made of 20.3 cm aluminum flashing, but rings of this size were too large for the established plots. To accommodate two rings in each of the 3 m diameter plots, the ring diameter was reduced to 37.5 cm. Height was proportionally reduced 25% to 15.2 cm to prevent over-shading. Slug exclosure rings were rimmed at the top with a 5 cm wide band of copper tape as copper provides a repellant effect on slugs [37]. The control rings lacked the copper tape and had three 4×8 cm sections removed from the base to allow slug movement in and out of the ring. The rings were installed in the plots on August 5, 2011 (S1 Fig). The rings were fastened flush to the ground with several stakes made from bailing wire. There were two rings per plot (one exclosure and one control) for a total of 40 rings. The aboveground plant biomass was also harvested from within the rings during the tri-annual harvests of the field. Slug herbivory was quantified as the difference in aboveground plant biomass between each set of paired collars within a plot. If slug exclosures had more plant biomass measured than the adjacent control ring, then slug herbivory had occurred at the measured level of consumption. If the two rings were equal in plant biomass or biomass of the slug exclosure exceeded the control, then no discernible slug herbivory was detected.

### Slug abundance

Live traps were set at regular intervals during the final year of study to quantify differences in slug abundance between the climate treatments. From several preliminary trials (unpublished data), it was determined that the most reliable method of live trapping was to place a 100 mm diameter petri dish with ~10 g of wet cat food (Friskies Classic Paté, Nestle Purina Petcare Company, St. Louis, MO, U.S.A.) on the ground surface in a plot for 15 minutes just after dawn. This method attracted the greatest number of slugs consistently and is similar to ‘saucer trapping’ in that the area from where the slugs originate is unknown. Therefore, slug density could not be calculated [38]. Trapping was conducted every two weeks from January 3, 2013, until October 10, 2013. Slugs were released in the plot from which they were trapped.

Preliminary methods testing over six months (unpublished data) revealed the importance of concluding the trapping no later than 45 minutes after sunrise, as catch efficacy of the traps was significantly reduced after this time. Starting at sunrise, baited dishes were placed randomly in each of the four plots within the first of five blocks and left for 15 minutes. Baited dishes were placed in the second experimental block 5 minutes after dishes were placed in the first block. At any time, two blocks were being trapped so that their 15-minute periods were on a staggered schedule, allowing for all 5 blocks to be trapped within 45 minutes. The order in which blocks were trapped was determined by a random sequence generated by PROC PLAN in SAS (SAS Institute Inc. 9.3, Cary, NC, U.S.A) the day of each trapping event. After 15 minutes, each dish was examined, and the length, number, and species of each slug on the dish was recorded.

Additional slug trapping occurred outside the experimental plots from August 15 to October 15, 2013. These slugs were taken to the laboratory and used to quantify the relationship between slug length and dry biomass. Slug length was measured in the same way as occurred during plot trapping and then slugs were dried in a 105°C forced air drying oven for 24 hours and weighed. The length versus biomass curve was used to estimate the biomass of slugs caught during trapping so that it was only necessary to measure the length of slugs when live-trapping as this was a time sensitive process. The curve was determined using the model ln(mass) = A + B*ln(length) in SAS using PROC REG as discussed in Hawkins, Lankester, and Lautenshlager [39]. The model yielded the equation: mass(mg) = 0.007*length(mm)^2.92^ with an R^2^ value of 0.5855 (*p* = 0.0099, n = 10, S2 Fig). The majority of slugs captured (84%) had lengths that fell within the range of the curve used to estimate mass (S3 Fig).

### Forage quality & plant community composition

Carbon and nitrogen (%C and %N) analysis (FlashEA 1112 NC Soil Analyzer, ThermoFisher Scientific, Waltham, MA, U.S.A.) was performed on a random grab sample of forage taken from each plot after it had been mowed across the study period. Visual cover estimates were recorded by species (to 1%) for each slug exclosure and control ring (n = 40) immediately preceding the final harvest in October 2013.

### Statistical analysis

Slug herbivory, slug count and biomass, and plant biomass quality across climate treatments were analyzed as a 2×2 climate treatment factorial (heat×precipitation) with the lme4 package in R Studio (Version 4.2.0; [40]). For all models, heat and precipitation climate treatments were fixed effects. The slug herbivory models included additional fixed effects of exclosure and time, and the biomass quality models also had a fixed effect of time. Block and its interaction with the highest order interaction (i.e., heat×precipitation in the slug count model or heat×precipitation×treatment in the slug herbivory model) were specified as the random terms to properly calculate of degrees of freedom. If a random term returned an estimated variance of 0 (‘boundary is singular’), the random term with an estimated variance of 0 was removed from the model. Type III Analysis of Variance with Satterthwaite’s method was used. Pairwise comparisons were conducted on significant effects (*p* < 0.05) with emmeans.

Differences in aboveground plant biomass data for modeling slug herbivory were normally distributed as determined by the Anderson-Darling test on four of seven harvest dates. The three harvests which were not normally distributed were determined to be within tolerances of an ANOVA model [41], so no transformation was performed. Slug number was square root transformed. The square root of count and the estimated mass of slugs trapped were not normally distributed but were determined to be appropriate for analysis by ANOVA by visual assessment of histograms [41]. On dates in which no slugs were trapped, no mass, rather than a mass of 0, was used for statistical analysis. Zero values were retained in count data.

Plant species cover data taken from the slug exclosure and control rings prior to the October 2013 harvest were analyzed using non-metric multidimensional scaling ordination in PCORD (NMS; PCORD version 6.0, MjM Software, Gleneden Beach, OR). Pairwise comparisons of the climate treatment plant species cover estimates were performed with MRPP (multi-response permutation procedures) analysis, and p-values were corrected using the Bonferroni-adjustment for the number of comparisons (α = 0.05/6: α_adj_ = 0.008).

## Results

### Effect of climate on slug herbivory

**Fig 2:**
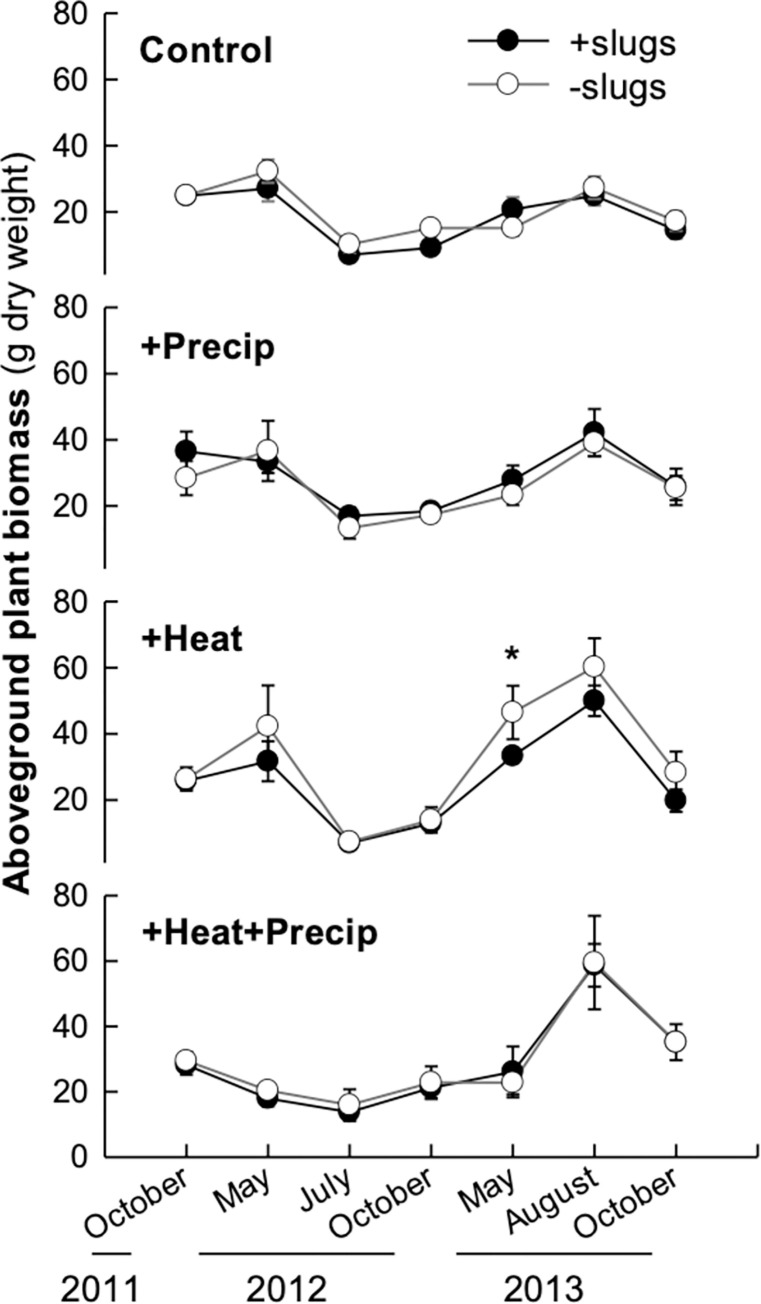
Average (± S.E.) aboveground plant biomass harvested from the control and slug exclosure rings for each climate treatment over the seven harvest dates. The asterisk denotes a significant difference in plant biomass between the control and slug exclosure rings in the heated plots only in May of 2013.

**Fig 3:**
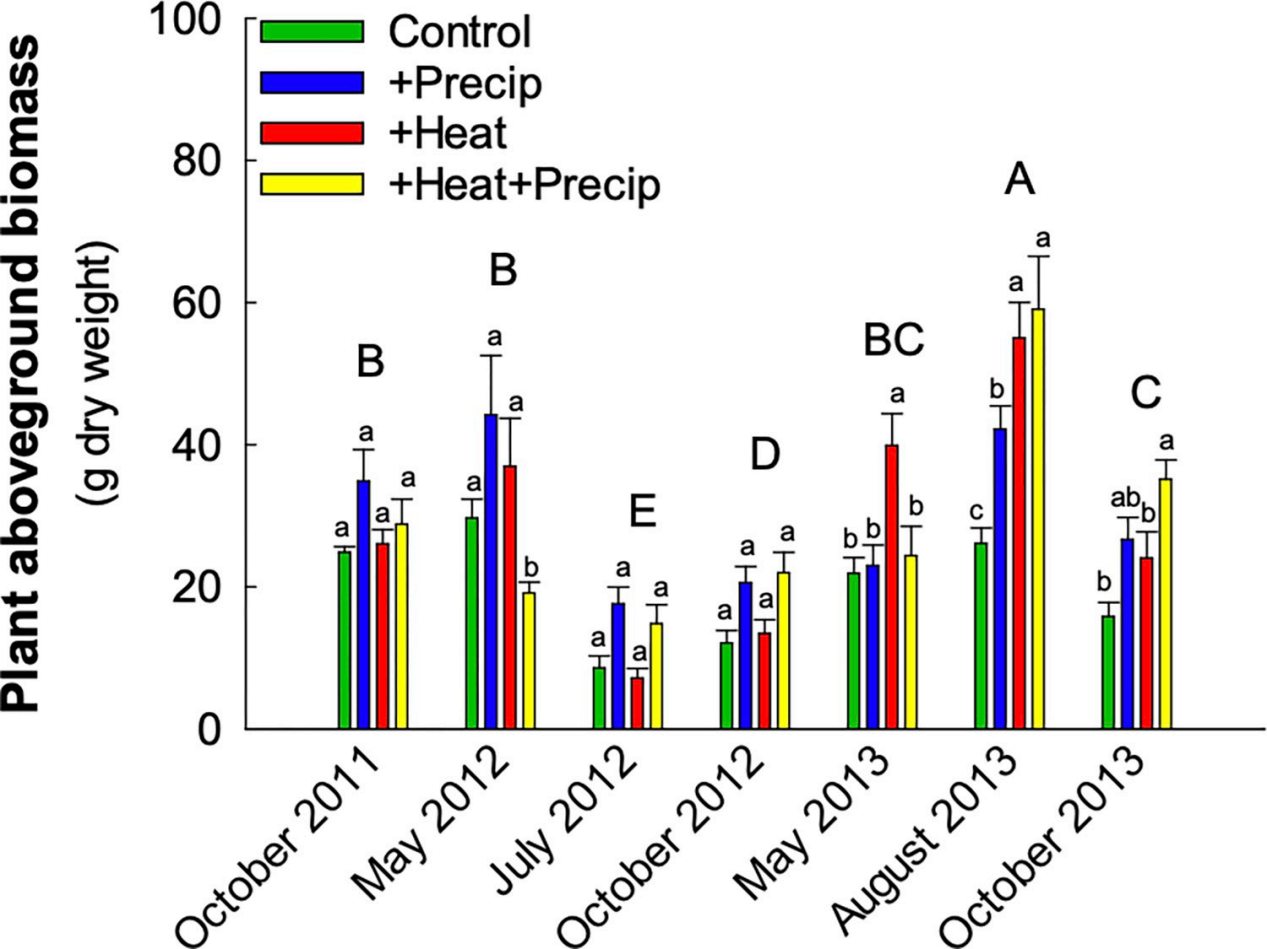
Average (± S.E.) plant biomass per ring harvested in each climate treatment from October 2011 to October 2013. Capital letters denote significant differences across the harvests, and lowercase letters denote significant across climate treatments within each harvest.

Over the entire two-and-a-half-year duration of the study, slug exclusion had no overall effect on harvested aboveground plant biomass (Table 1; Fig 2). On the May 2013 harvest date, there was a significant effect of slug exclusion in the heated plots with greater plant biomass harvested from the slug exclosure rings than the control rings. May 2013 was the only harvest during the study in which the heat treatment had significantly more total plant biomass than the other climate treatments (Fig 3). Seasonal variation in harvested plant biomass was not consistent across years, with the least amount of plant biomass occurring in July 2012 (11.3 ± 1.1g average plant biomass per ring) and the greatest amount in August 2013 (45.5 ± 3.3g plant biomass; Fig 3). While additional precipitation tended to increase plant biomass at most harvests, the effect was only significant in Fall 2013 (Fig 3). In May 2012 & 2013, additional precipitation significantly reduced plant biomass in the combined climatic treatment (+heat+precipitation) compared to the +heat alone.

**Table 1:** ANOVA results illustrating the effect of slug exclusion on aboveground plant biomass within the climate treatment plots over time. Degrees of freedom are shown as numerator, denominator. Bolding indicates significant main effects and interactions.

Effect	DF	F-Value	P-Value
**heat**	1,30	4.88	**0.0349**
precip	1,30	2.54	0.1210
heat×precip	1,30	2.41	0.1313
exclosure	1,30	0.64	0.4290
heat×exclosure	1,30	0.45	0.5091
precip×exclosure	1,30	1.36	0.2530
heat×precip×exclosure	1,30	0.00	0.9965
**time**	6,180	60.46	**<0.0001**
**heat**×**time**	6,180	11.68	**<0.0001**
**precip**×**time**	6,180	6.33	**<0.0001**
**heat×precip×time**	6,180	3.53	**0.0025**
exclosure×time	6,180	0.58	0.7447
heat×exclosure×time	6,180	0.21	0.9720
precip×exclosure×time	6,180	0.32	0.9252
heat×precip×exclosure×time	6,180	0.52	0.7945

### Effect of climate on slug abundance and biomass

**Fig 4:**
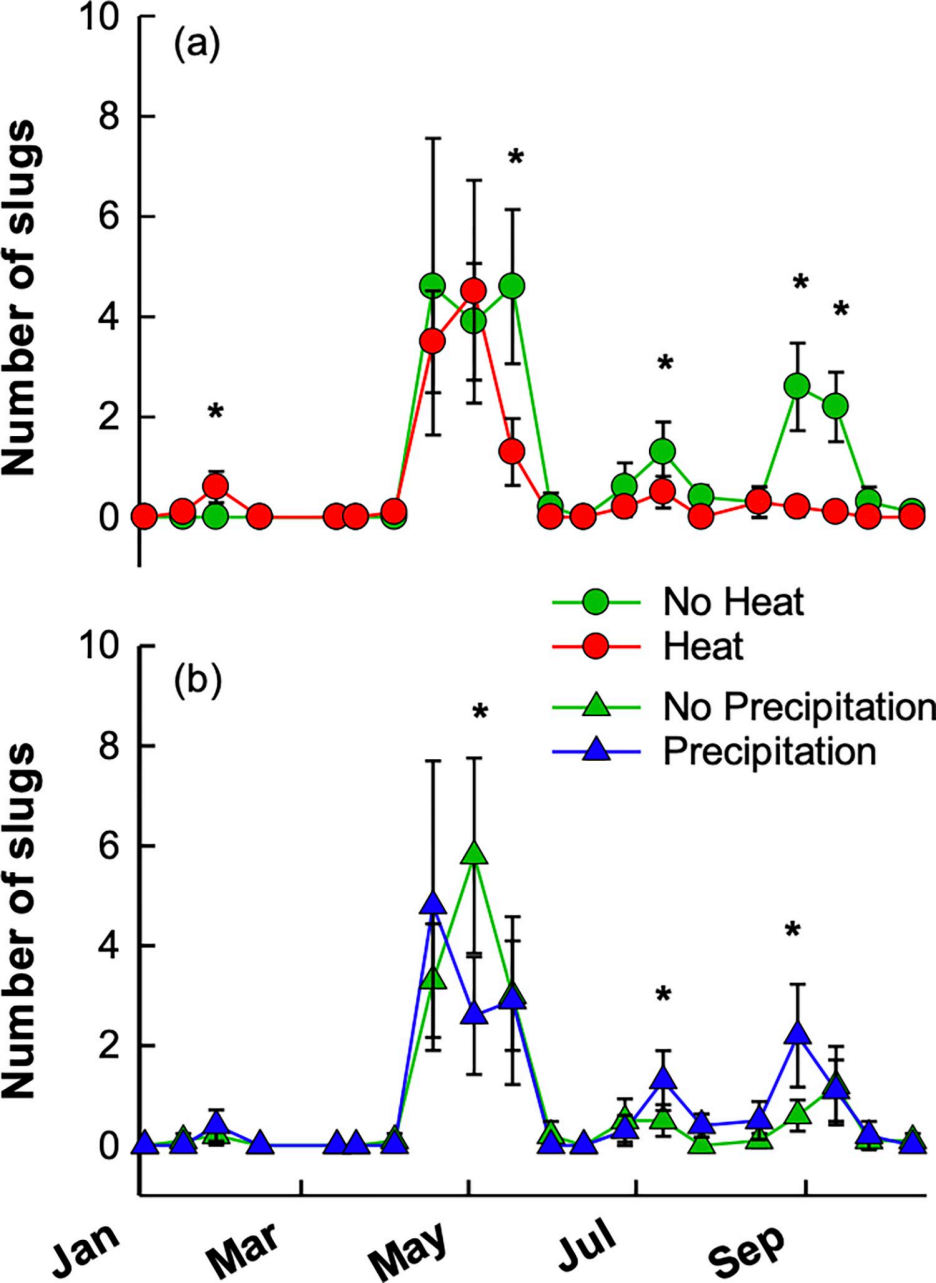
Average (± S.E.) number of slugs trapped in heated vs. non-heated plots (a) or in added precipitation vs. ambient precipitation plots (b). Points in time where significant climate treatment effects were observed are indicated by asterisks. Fescue growing season was March 15-October 15 with harvests occurring mid-May, early August, and mid-October. Seasons are defined as: winter—December-February, spring—March-May, summer—June-August, and fall—September-November.

Slug count, but not biomass, was significantly reduced in heated vs. non-heated treatments, but this climate treatment effect was modified by time (Tables 2 and 3). At one sampling date in winter, heated plots had greater slug populations than non-heated plots (average of 0.6 v. 0 slugs), but the heat effect was reversed in the late spring, mid-summer, and early fall in which control plots contained 62–95% more slugs (Fig 4). A significant time×precipitation interaction was also apparent for slug counts. Plots receiving additional precipitation had more slugs than plots that received no additional precipitation but only in mid-summer and early fall, but this trend was reversed on a single day in May (Fig 4). Overall time effects were apparent, as number of slugs trapped was greater in late spring and early fall than throughout the rest of the experiment (Fig 4). Slug species was not considered in the analysis as only 4 of 325 slugs trapped in 2013 (1.2%) were not *D*. *reticulatum*. *Arion hortensis* (Férussac) was the only other slug species present and occurred in both control and precipitation plots, albeit very rarely.

**Table 2:** Average number and estimated biomass (± S.E.) of slugs caught per plot by climate treatment across all 20 trapping events. Letters indicate significant differences across climate treatments.

Treatment	Number of Slugs	Biomass of Slugs (mg)
Control	0.92 ± 0.19 a	45.65 ± 8.39 a
Precipitation	1.19 ± 0.28 a	69.14 ± 11.01 a
Heat	0.66 ± 0.21 b	59.41 ± 15.36 a
Heat **×** Precipitation	0.48 ± 0.13 b	44.15 ± 12.59 a

**Table 3:** ANOVA table for the average count and biomass of slugs trapped per plot for 2013 in the climate change treatments. Degrees of freedom are numerator, denominator. Bolding indicates significance.

	Slug Count			Slug Biomass		
Effect	DF	F	P	DF	F	P
**Heat**	1,12	11.74	**0.0050**	1,62	0.27	0.6058
Precip	1,12	0.15	0.7055	1,63	0.08	0.7836
Heat × Precip	1,12	0.90	0.3603	1,63	0.59	0.4438
**Time**	19,304	23.52	**<0.0001**	14,62	0.49	0.9322
**Time × Heat**	19,304	3.89	**<0.0001**	7,61	1.83	0.0974
**Time × Precip**	19,304	1.67	**0.0407**	9,61	0.71	0.6999
Time × Heat × Precip	19,304	1.44	0.1059	5,60	0.48	0.7919

### Forage quality

**Fig 5:**
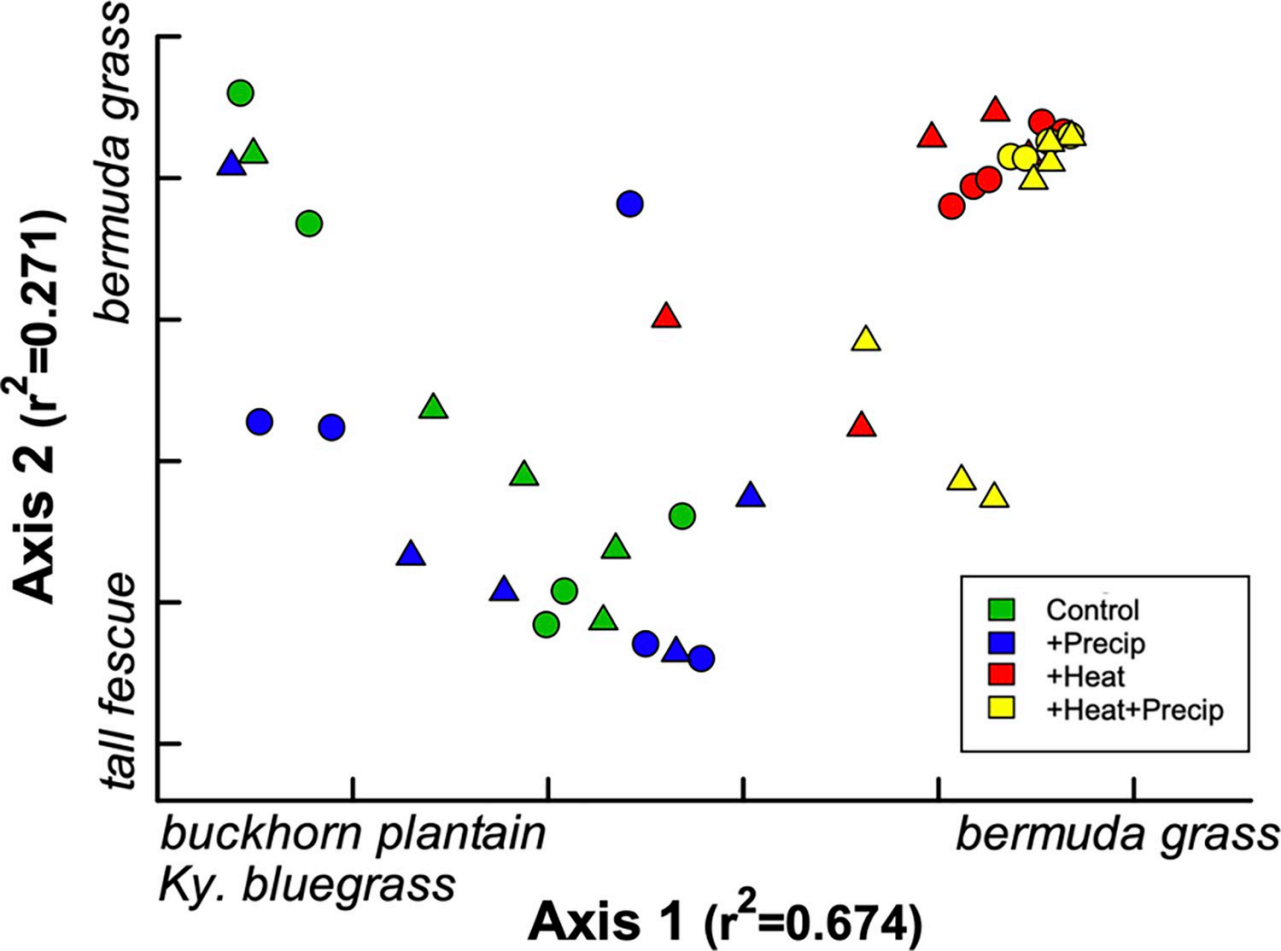
Ordination of plant species cover in the slug exclosures (triangles) and control rings (circles) from each climate treatment plot. The plant species noted at each end of the axes indicate the positive and negative drivers of the ordinations and had an R-value ≥ 0.65.

Ordination analysis of the slug exclosure and control ring plant cover estimates showed two main groupings, which were defined by the heat treatment. Heated plots had distinctly different vegetative communities compared to the non-heated plots at the October 2013 harvest (Fig 5). Heated plots had greater abundance of the warm-season (C_4_ physiology) Bermuda grass (*Cynodon dactylon* (L.) Pers), while non-heated plots were dominated by cool-season (C_3_) species—tall fescue (*Schedonorus arundinaceus* (Schreb.) Durmort), buckthorn plantain (*Plantago lanceolata* L.*)*, and Kentucky bluegrass (*Poa pratensis* L.). Pairwise comparison analysis using MRPP confirmed this result, yielding significant differences in all comparisons between heated and non-heated treatments (S1 Table). There was no grouping that suggested differences between control and slug exclosure rings, illustrating that slug exclusion for >2 years had no detectable effect on the plant community composition.

**Fig 6:**
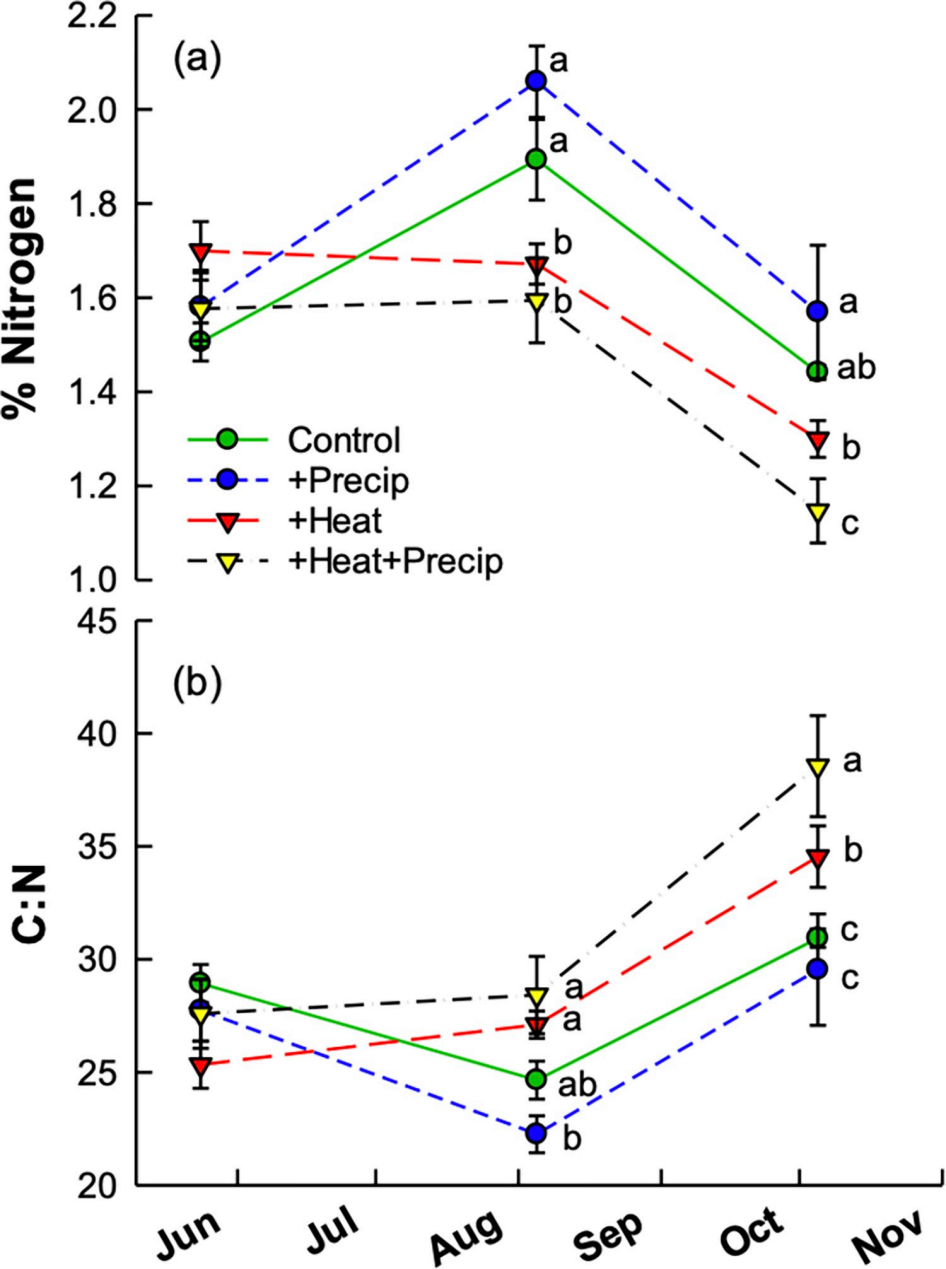
Average %N (a) and C:N (b) (± S.E.) of bulk forage harvested from the climate treatments over the 2013 growing season. Letters indicated significant differences within a given harvest date. There were no significant differences across climate treatments on the first harvest date. Fescue growing season was March 15-October 15 with harvests occurring mid-May, early August, and mid-October. Seasons are defined as: winter—December-February, spring—March-May, summer—June-August, and fall—September-November.

Significant effects of heat, heat×precipitation, time, and heat×time were observed for C:N ratios of grab-sampled forage for the year in which slugs were trapped (Table 4). Heated plots had higher average C:N values (± S.E.) than non-heated plots (30.3 ± 1.0 v. 27.3 ± 0.7, respectively). However, the effect of heat was only significant in summer and autumn harvests (Fig 6) when the plots were dominated by C_4_ grasses (unpublished data). The addition of precipitation to +heat and ambient (control) plots affected C:N differently. In +heat+precipitation treatments, C:N was subtly augmented compared to +heat alone; while in +precipitation treatments, C:N was slightly lower than in ambient control plots (Fig 6). In the spring, when plant community composition was more similar across climate treatments, C:N ratios did not differ (Fig 6).

**Table 4:** ANOVA table for C:N and %N of bulk forage samples from the 2013 harvests. Degrees of freedom are shown as numerator, denominator. Bolding indicates significance.

	C:N			%N		
Effect	DF	F	P	DF	F	P
**Heat**	1,44	13.88	**0.0006**	1,12	16.30	**0.0016**
Precip	1,44	0.30	0.5837	1,12	0.00	0.9506
**Heat × Precip**	1,44	7.16	**0.0104**	1,12	7.55	**0.0177**
**Time**	2,44	36.20	**<0.0001**	2,32	39.33	**<0.0001**
**Time × Heat**	2,44	9.92	**0.0003**	2,32	11.42	**0.0002**
Time × Precip	2,44	0.47	0.6277	2,32	0.28	0.7636
Time × Heat × Precip	2,44	0.15	0.8584	2,32	0.09	0.9153

## Discussion

Despite prior work illustrating that slug herbivory can significantly reduce plant production in pastures [1, 16] and our hypothesis that it would do so at this site, we found little-to-no evidence that slugs impact plant production or plant species composition in our central Kentucky pasture. Overall, our results indicate that slug herbivory is relatively limited at this site, despite catching 325 slugs during the 2013 growing season.

In addition to eating aboveground plant material, slugs consume litter and detritus [42]. This is a possible explanation for why few significant effects on live aboveground plant biomass were recorded. Slugs at this site may have primarily consumed detritus, which we did not measure. Previous research on the effects of slug exclusion has focused mainly on establishment and development of plants, finding that slugs can influence the development of pastures and other ecosystems through seedling herbivory [1, 27, 43, 44]. Because the pasture for this project was established with perennial species more than three years prior to the beginning of this experiment, it is possible that the primary influence of slugs on seedling survival and its subsequent impacts on the resulting plant community dynamics was missed. Several plant species present in the pasture were annuals (comprising on average 18% of the biomass), sprouting new seedlings each spring and autumn. Still, few effects of slug herbivory were observed, suggesting overall effects were negligible.

We hypothesized that seasonal trends in moisture and temperature would interact with the imposed climate treatments to alter effects of slug herbivory and influence slug abundance and slug biomass. We found that seasonal differences in slug abundance were modified by the climate treatments in ways that we predicted. When moisture was limiting (in late summer), +heat treatments were hotter and drier than non-heated plots (Fig 1) and had lesser numbers of slugs (Fig 4). This result is consistent with warming-associated reductions in slug abundance at Jasper Ridge Biological Preserve [45]. At our site, added precipitation increased the abundance of slugs during the late summer. While we did not monitor or measure slug activity, slugs may have been more active during this time period. Slug abundance varied across the year. Slugs were present aboveground in significant numbers during the spring, early summer, early autumn, an unseasonably warm week in February, and an unseasonably cool and wet period between mid-June and early July, illustrating, as others have reported, that slugs are sensitive to the temperature and moisture levels in the field [12, 22, 23].

We additionally hypothesized that seasonal changes in temperature and moisture would interact with the climate treatments to alter plant community composition, forage quality, and biomass. We found evidence that warming significantly modified plant community dynamics and forage quality. While the abundance of C_4_ species and C:N ratio increased overall in all treatments as the season progressed, +heat treatments exhibited greater increases compared to the other treatments. This is consistent with research suggesting that warming will reduce overall forage quality [46]. Heat, precipitation, and their interaction modified the seasonal effects on plant biomass. Plant biomass seasonal changes and climate modifications were inconsistent across the years, illustrating that the effects of climate change are subject to inter-annual variation, a result supported by others [47, 48]. For example, during the drought in 2012, the +precipitation treatments had greater biomass than the control plots, but during the relatively wet year of 2013, the +heat treatments had significantly greater biomass. Climate change may make inter-annual variation in harvestable biomass more variable, which is consistent with predictions by White et al. [46].

We further hypothesized that increased heat, increased precipitation, and their combination would allow persistence of slugs during times of year when temperature or moisture were not conducive to slug survival. Consistent with this hypothesis, we found that slugs persisted in +heat treatments during the winter and in +precipitation treatments during the summer. The effect was particularly evident in winter 2013 when slugs were more abundant in +heat plots. If similar effects occurred in winter 2012 (unfortunately, we did not trap slugs in 2012), this may have resulted in the only significant slug herbivory being observed occurring in the +heat treatment in the spring of 2013. Heat effects observed in slug abundance were also observed in forage quality metrics. Greater slug abundances in non-heated plots at the end of the summer coincides with the largest difference in C:N between heated and non-heated plots. During the late summer and fall, heated plots primarily consisted of C_4_ grasses, contrasting with C_3_ dominant grasses in ambient plots, explaining the large difference in C:N of the bulk forage for those plots. In addition, research has shown that invertebrate herbivores prefer C_3_ grasses to C_4_ grasses [49, 50]. It is possible that the greater abundance of slugs in non-heated plots was related to the significantly higher quality of available forage, though it should be noted that no significant effect of slug exclusion (i.e., herbivory) was measured at this time.

It appears that while the climate treatments altered both slug abundance and forage quality in late summer and early fall, the ecological impact of the slugs as grazers remained unchanged. These findings illustrate that the climate treatments can indirectly influence slug abundance through changes in plant community dynamics and forage quality. However, it is important to note that there was a significant effect of slug herbivory in the heated plots during the spring of 2013 when the differences in plant community and forage quality were minimal (unpublished data), suggesting that climate treatments were also directly influencing slug activity. Increasing temperatures have been reported to increase the herbivory of *D*. *reticulum* by reducing the presence of *D*. *reticulum*’s nematode parasite, *Phasmarhabditis hermaphrodita [51]*. *Phasmarhabditis hermaphrodita* is not common in the United States, but it is possible that temperature impacts on slug predator species could result in changes in slug herbivory patterns, such as that found in this study.

## Conclusions

Slugs did not have an impact on plant biomass production and community dynamics in this hay-managed pasture of central Kentucky under current climatic conditions or the imposed climate treatments (e.g., increased temperatures and additional precipitation), except in the early growing season when slug herbivory and abundance were directly stimulated by increased temperatures. Later in the season abundance appeared to be related to both direct and indirect effects of the imposed climate treatments. Increased precipitation buffered the negative impacts of hot and dry conditions in late summer and early fall on slug abundance, but differences at this time of the year between heated and non-heated plots may have been related to a direct negative effect of heat on slugs and/or a decrease in plant %N and forage quality associated with heat-driven changes in the plant community. Although slug abundance was sensitive to abiotic and biotic changes resulting from the climate change treatments, the limited effect of slug herbivory measured with exclosures warrants greater study. Impacts of climate change on slug herbivory were not observed in this study, likely due to low slug abundance, as others have found changes in slug herbivory with climate [12, 15, 27]. In areas where slugs are more abundant or in grasslands dominated by annual species where seedlings are abundant each year [45], the effect of climate change on slug herbivory may be more pronounced and important for continued ecosystem function.

## Supporting information

S1 Table Pairwise comparisons of the plant species cover estimates for the climate treatments done with MRPP analysis in the ordination, showing that the plant community of all heated plots differed significantly from that of the non-heated plots. Bolding indicates significance.
(PDF)

S1 Fig Paired slug control (left) and exclosure (right) rings within an experimental climate treatment plot (photo by D. Weber). (TIF)

S2 Fig Relationship between slug length and mass developed from *Deroceras reticulatum* caught on-site and used to convert slug abundance data from trapping in the treatments to mass estimates. (TIF)

S3 Fig Histogram of the number of slugs of each size that were trapped during the 2013 season. Sizes range from 2.5 mm to 35 mm.
(TIF)
