# Supplementary material for: Climate change alters slug abundance but not herbivory in a temperate grassland
Source: PLoS One. 2023 Mar 14;18(3):e0283128. doi: 10.1371/journal.pone.0283128 (PMC10013886; doi:10.1371/journal.pone.0283128)
Supplement: S1 Table — Bolding indicates significance. (PDF) [file pone.0283128.s001.pdf]

Comparison	*p*-value
**+Heat+Precip vs. +Precip**	**0.000012830**
+Heat+Precip vs. +Heat	0.061953540
**+Heat+Precip vs. Control**	**0.000007520**
**+Precip vs. +Heat**	**0.000044430**
+Precip vs. Control	0.450131430
**+Heat vs. Control**	**0.000020670**
