# Supplementary figures and images for: Climate change alters slug abundance but not herbivory in a temperate grassland
Source: PLoS One. 2023 Mar 14;18(3):e0283128. doi: 10.1371/journal.pone.0283128 (PMC10013886; doi:10.1371/journal.pone.0283128)

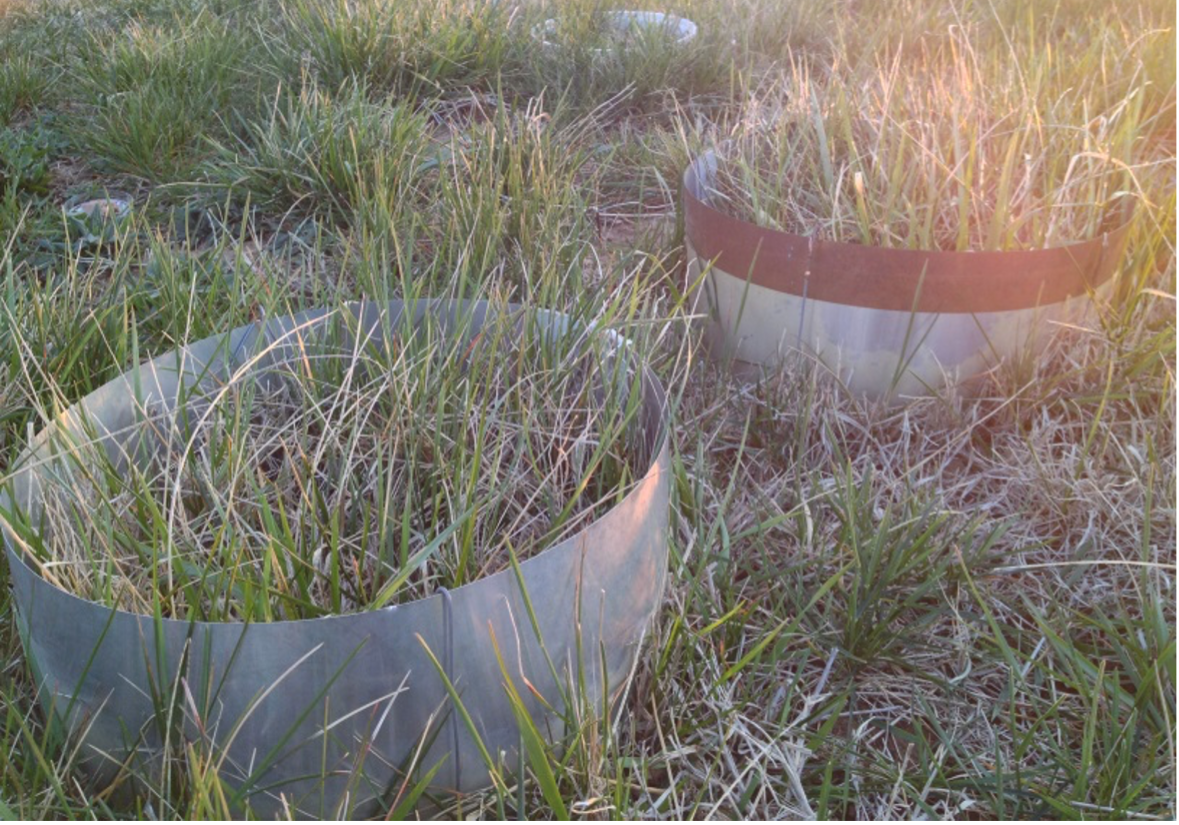

Supplement: S1 Fig — (TIF) [file pone.0283128.s002.tif]

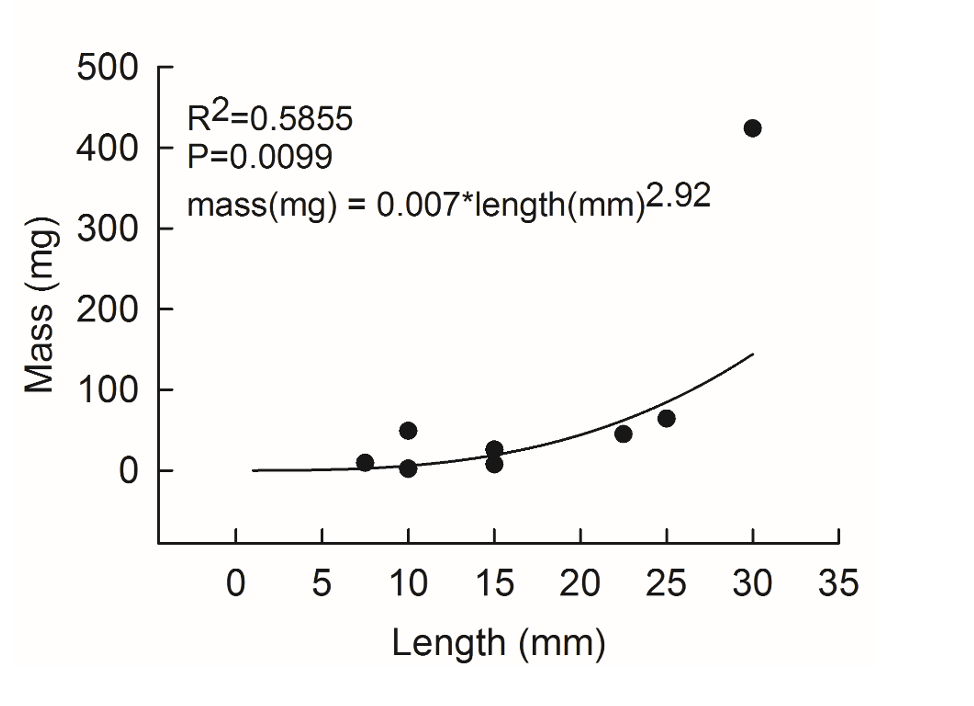

Supplement: S2 Fig — (TIF) [file pone.0283128.s003.tif]

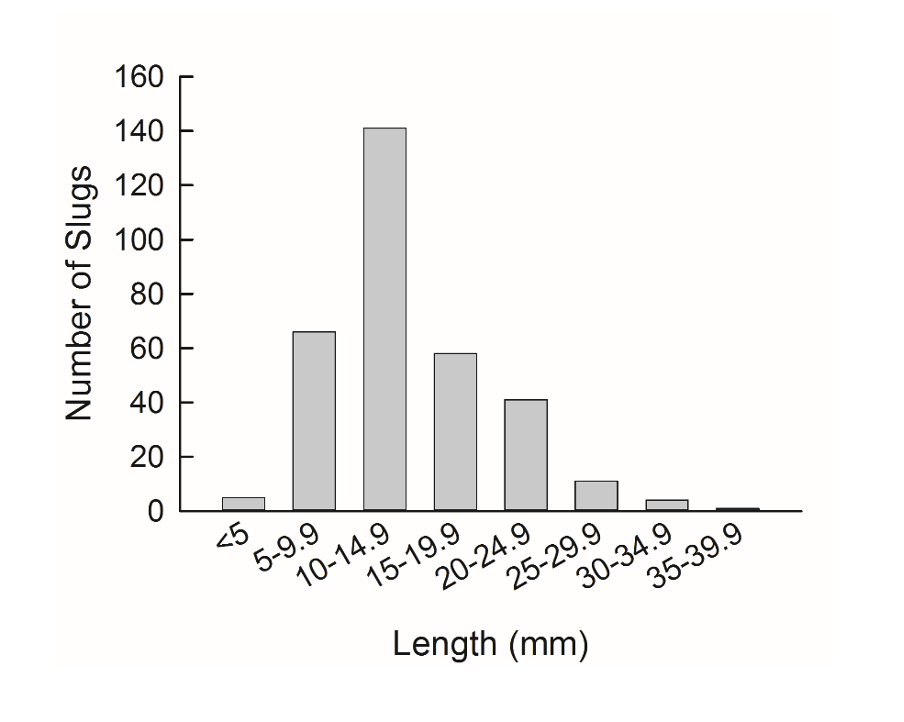

Supplement: S3 Fig — Sizes range from 2.5 mm to 35 mm. (TIF) [file pone.0283128.s004.tif]
